# A Comparative Evaluation of Microimpedance Tomography Reconstruction Algorithms for in Vitro Imaging

**DOI:** 10.1021/acssensors.5c03758

**Published:** 2025-11-06

**Authors:** Chang Liu, Xingyang Chen, Thomas E. Winkler, Iordania Constantinou

**Affiliations:** † Institute of Microtechnology (IMT), 26527 Technische Universität Braunschweig, Alte Salzdahlumer Straße 203, 38124 Braunschweig, Germany; ‡ Department of Micro and Nanosystems (MST), KTH Royal Institute of Technology, Malvinas väg 10, 100 44 Stockholm, Sweden; § Digital Futures, KTH Royal Institute of Technology, Malvinas väg 10, 100 44 Stockholm, Sweden; ∥ Center of Pharmaceutical Engineering (PVZ), 26527 Technische Universität Braunschweig, Franz-Liszt-Str. 35a, 38106 Braunschweig, Germany

**Keywords:** electrical impedance tomography, image reconstruction, machine learning, microfabrication, zebrafish monitoring

## Abstract

This paper presents the development of a novel miniature electrical impedance tomography (EIT) system made out of glass, along with the training, validation, and testing of an accompanying open-source machine learning image reconstruction model. Our 1-dimensional convolutional neural network (1D-CNN) models were uniquely benchmarked, both qualitatively and quantitatively, using synthetic and experimental data, against well-established image reconstruction methods: the one-step Gauss–Newton method and the total variation reconstruction method. Image reconstruction results obtained using our 1D-CNN show significant advantages over these traditional methods, achieving an up to 5-fold reduction in mean square error on synthetic data. These results were replicated for two common excitation/measurement modes and extended to objects with varying conductivity and quantities. The superior EIT reconstruction capabilities of our 1D-CNN were further validated experimentally across a similarly broad range of parameters, achieving an average positional accuracy of 147 μm and an average dimensional resolution of 70 μm. To demonstrate potential applications in in vitro monitoring, we used our platform to observe zebrafish development through three distinct phases, from embryo to larvae, showcasing our platform’s compatibility with biological imaging.

## Graphical abstract

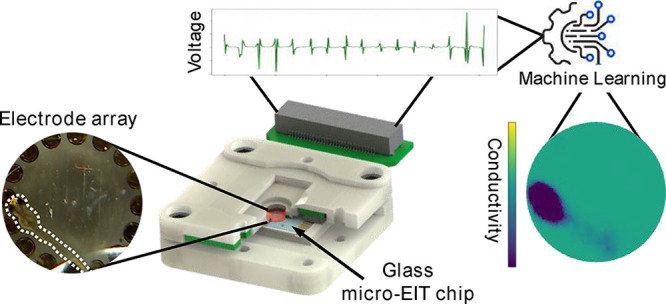

Electrical Impedance Tomography (EIT) is an imaging technique that reconstructs images of the internal conductivity distribution within a measurement zone by applying small electrical currents through multiple electrode pairs and measuring the resulting voltages. This unique imaging method has significant application potential in biology and medicine, being cost-effective, noninvasive, and label-free.^1,2^ Henderson and Webster first considered its use in the analysis of respiration.^4^ To capture respiratory patterns, they applied a 100-electrode array to a subject’s back and a flexible plate electrode to the subject’s chest to establish an equipotential surface onto which the voltage source was connected. Nowadays, respiratory monitoring using EIT is achieved by strategically positioning electrodes in a transverse layout encircling the patient’s chest. Fluctuations in lung volume during breathing result in measurable conductivity changes due to the different conductivities of air and lung tissue.^5^

In recent years, there has been a growing effort to develop miniaturized EIT implementations for biomedical research. Part of this concerns imaging tissue in live subjects smaller than humans. For example, Aristovich et al. utilized EIT through epicortical arrays with 30 electrodes to image electrical activity in the mouse brain (with resolution below 200 μm and 2 ms).^6^ Another, equally, if not even more impactful, application field is the monitoring of biological in vitro culture systems. Optical microscopy, the primary method for cell and tissue characterization, is often invasive and may interfere with the (patho-)­physiological conditions to be studied. EIT can be used as a noninvasive alternative to provide insights into cell growth and responses, thus greatly advancing the progress of precision medicine and drug development. The first implementation of miniaturized EIT for cell culture monitoring came from Lemmens et al., who employed a ring-shaped micro-EIT setup with 20 mm diameter and eight electrodes to monitor yeast cultivation.^7^ Subsequently, Yin et al. introduced a novel dual-ring EIT sensor featuring 16 measurement electrodes and a diameter of 12 mm. This sensor was specifically designed for 3-dimensional imaging of cell aggregates and investigating cellular responses to drugs.^8^ Despite the advancements in miniaturization, both studies suffer from some obvious limitations, emblematic also of other work in the field:^9^ First, it is estimated that EIT resolution in setups utilizing ring-shaped electrode configurations roughly scales as 10% of the array diameter;^10^ imaging resolution was thus in the mm-scale in both studies. The highest-resolution EIT array to date was likely that of Li et al., who developed a ring-shaped EIT sensor with a mere 150 μm diameter for single-cell (MSG-C6 cell) imaging. This was, however, not intended for cell culture monitoring, but rather as a tool for flow-through cell analysis building upon the field of impedance cytometry. Its adaptability to other application areas was further limited by the use of copper electrodes (lack of biocompatibility) on a PCB (interfering with optical observation). The second limitation of current works lies in image reconstruction, a nontrivial computational challenge. Standard software toolkits, such as the open-source PyEIT^3^ and EIDORS,^29^ are commonly employed for image reconstruction; however, contrast is often poor. The impact of algorithmic choice and reconstruction parameters on imaging resolution becomes apparent, for instance, in Lemmens’ study in which different algorithms were compared.^7^ Alternative proprietary/closed-source software (such as the one used in Lemmens’ study^7^ or mentioned by Alanis et al.^11^) rarely perform better (though benchmarking is practically nonexistent) and introduce additional constraints, notably limited customization and delayed updates, which can hinder the adaptation to new research developments and specific imaging needs.

There have naturally been a number of recent advances in EIT reconstruction over the previous years across application domains, from geophysical to structural to biomedical.^12^ One example concerns regularization, wherein the reconstructed conductivity distribution is constrained by minimizing a specified mathematical norm of the solution, damping the magnification of measurement errors. Whereas traditional l2 -norm approaches favor smoothness, l1 -norm and mixed regularization have shown great advantages in reconstruction of sparse or sharply delineated objects or defects.^13^ Toward a similar end goal, others have pursued more explicit consideration of shape and conductivity priors in the reconstruction.^14^ Yet other lines of research have introduced Bayesian concepts to make reconstruction more robust albeit more computationally complex and intensive.^15,16^

Most recently, EIT image reconstruction with the help of supervised machine learning (ML) has received the bulk of the attention. For example, Li et al. trained an image reconstruction model based on a 1-dimensional Convolutional Neural Networks (1D-CNN),^17^ and Wei et al. leverage a CNN in their Dominant-Current Deep Learning pipeline to improve both accuracy and computational efficiency over a more classical iterative solver.^18^ Both studies demonstrate good reconstruction performance (at 30 dB synthetic SNR, relative image errors of 6.5 and 9%), but lack benchmarking against standard methods, are aimed at human chest scale, and only utilized nonbiological phantoms for their experimental validation. Chen et al. extended the application of deep learning to the millimeter scale by employing a 14 mm diameter EIT array for imaging biological samples, specifically human breast cancer cell aggregates (2 mm diameter). They demonstrate improved performance against a selection of alternative algorithms, albeit marginal (at 30 dB synthetic SNR, image correlation coefficients of 56%, versus 50% for the worst algorithm included).^19^ While significantly larger improvements were demonstrated with experimental data, neither of the two reconstructions they evaluated included cell clusters located near the center of the array. In the field of structural monitoring, Zhao et al. highlighted the potential of such methods to overcome typical resolution limitations.^20^ In purely synthetic testing, their CNN achieved a resolution of ∼1% (relative to array dimensions; 85 dB SNR) for defect size and position within a solid-conductive matrix. Yet, overall, key limitations of software availability and quantitative algorithm benchmarking remain.

Given the remaining hardware and software challenges, we aim to develop the first micro-EIT chip compatible with both upright and inverted microscopy, as well as an associated digital framework for the reconstruction of images of biological samples for eventual in vitro culture monitoring. Specifically, we introduce a micro-EIT-on-chip platform featuring a 4 mm diameter electrode array on a transparent glass substrate. In parallel, we develop the first open-source imaging framework employing 1D-CNN to enhance the resolution of reconstructed images across the entire measuring area. In this paper we first introduce our micro-EIT platform, EIT measurement principles, and image reconstruction theory as well as model design. Then, using synthetic data, we present and discuss a comparative analysis between the image reconstruction results obtained using our machine learning models trained for different excitation and measurement modes, and traditional image reconstruction methods implemented in the existing open-source PyEIT and EIDORS packages. We moreover present experimental measurement and image reconstruction results for ∼1 mm-scale objects made of different materials, quantities, and positions within the array to obtain a uniquely comprehensive quantitative and qualitative benchmarking of both our 1D-CNN reconstruction framework and our micro-EIT system. Finally, we employ our platform to observe the development process of a millimeter-sized zebrafish egg to zebrafish larva over 72 h.

## Methodology

### Micro-EIT Chip Platform and Measurement

Our micro-EIT platform (Figure 1) consists, at its core, of a microfabricated 16-electrode ring array on a glass chip (array diameter 4 mm; electrode diameter 0.5 mm). The ring geometry reduces the computational load of image reconstruction compared to noncircular arrays.^21^ Additionally, together with the choice of glass as a substrate, it facilitates unhindered optical observation of 88% of the chamber. The number of electrodes is chosen to optimize resolution and reconstruction accuracy of EIT imaging without causing excessive computational burden.^22^ The electrodes are made of gold (on titanium adhesion layer) to ensure biocompatibility, and SU-8 photoresist is used to insulate and protect the electrode connection traces. A PCB board with spring contacts and an external cable interface is placed above the chip to connect with the instrumentation, with a 3D-printed holder ensuring alignment and defining the measurement chamber (Figures 1 and S1). While herein we rely on a top-view microcamera to facilitate benchmarking also for objects intersecting the electrodes, we emphasize that higher-resolution inverted imaging, often employed in cell culture monitoring, is equally possible. For further details on instrumentation and fabrication we refer to the SI.

**Figure 1:**
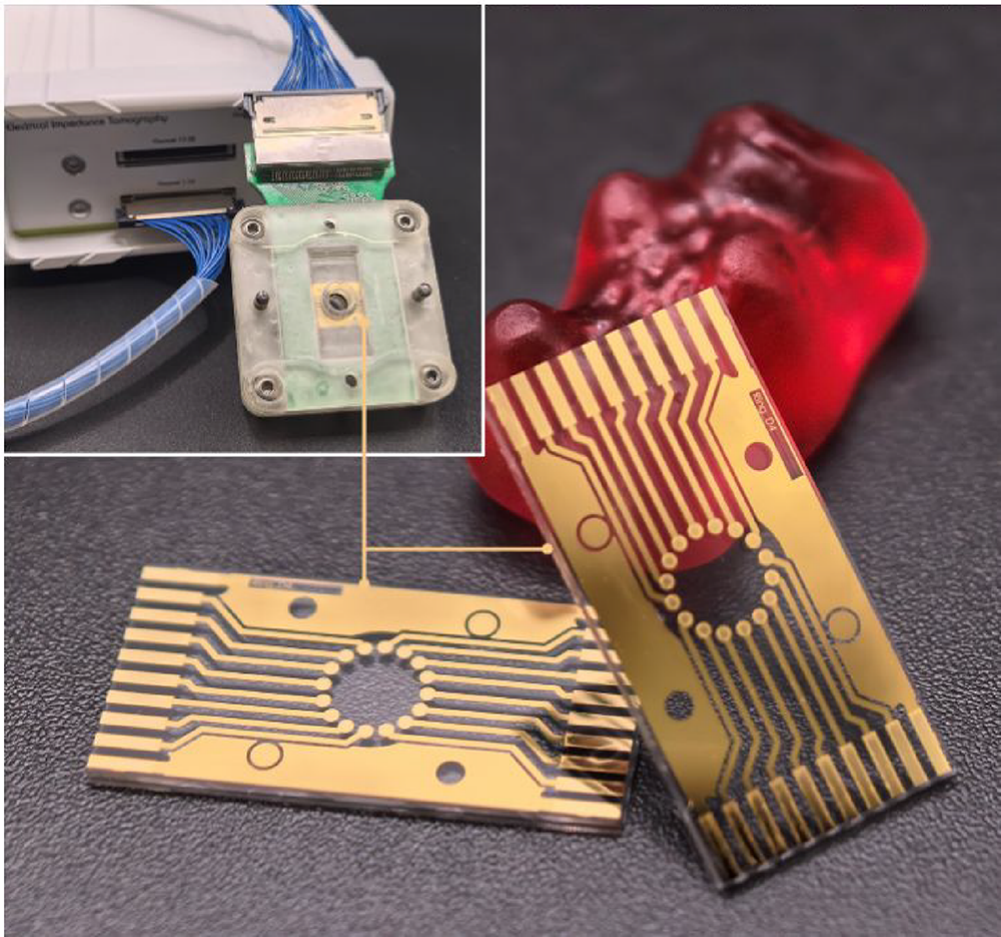
Micro-EIT platform. Our custom micro-EIT chip (10 × 20 mm^2^) features a ring-shaped array consisting of 16 gold electrodes on glass, with a measurement array diameter of 4 mm and an electrode diameter 0.5 mm (gummy bear for scale). Inset: The EIT instrumentation (Sciospec EIT32) connected to our device.

In the EIT measurement process, different excitation and measurement patterns describe the selection of electrode pairs used for current injection and the corresponding pairs used for voltage measurement. This is due to the prohibitive time required for (and limited benefit gained by) comprehensive mapping, which would require (*n* ^2^ – *n*)^2^/4 measurements for *n* electrodes. We specifically consider the adjacent injection – adjacent measurement pattern (ad–ad) and the opposite injection – adjacent measurement pattern (op–ad) (Figure S2). Whereas the ad–ad pattern should offer EIT sensitivity primarily near the electrodes, the op–ad pattern should improve EIT sensitivity in the central area of the measurement chamber.^23^ For the purpose of nondestructive and noninvasive measurement, a small injection current of 1 μA was used.^24^ The frequency of impedance measurements determines the depth and focus of cellular observations. At lower frequencies (typically below ∼100 kHz), the current is largely excluded from penetrating the cell membrane, making the measurement primarily sensitive to the external contour and boundary of the cell. In contrast, at higher frequencies (above several hundred kilohertz to a few megahertz), the current can pass through the membrane, enabling the detection of intracellular structures.^25^ Because we are primarily interested in the shape (outer contour) of the measured objects in the present study, a frequency of 10 kHz was chosen.^26^ All measurements take place in 0.3× Danieau solution (Table S2).

### EIT Image Reconstruction

The relation between the (many) individual measured voltages and the overall conductivity distribution within the chamber (i.e., the EIT image) can be described by a nonlinear but analytical function called the forward model. The model is “forward” because, given a conductivity distribution within an area of interest, it can predict measured voltages. The forward model is nonlinear because the relationship between conductivity and the resulting electric potentials is coupled with the geometric arrangement of the electrodes, the properties of the involved materials, and the applied currents. In order to simplify computations, this function is linearized; the result is a Jacobian matrix, which describes the sensitivity for an electrode array regarding certain current injection and measurement patterns.^1,25^ The objective of EIT image reconstruction is to solve the less determinate inverse problem, i.e., to use the recorded normalized voltages (more specifically, the voltage differences between the chamber containing the sample of interest and a corresponding empty chamber) to determine the conductivity distribution inside the imaging region.^27^ Traditional solvers can be broadly classified into smooth and nonsmooth methods depending on the properties of the typically applied regularization term.^28^ We thus consider two well-established methods in more detail: the one-step Gauss–Newton method (GN), as a representative smooth method and the total variation method (TV) as a representative nonsmooth method. GN involves a fixed Jacobian matrix (i.e., the derivative of the mapping function) and performs a single Gauss–Newton iteration on the Jacobian matrix to estimate the conductivity distribution.^27^ TV aims to preserve sharp changes in conductivity by penalizing excessive smoothness, and is implemented through more computationally intensive iterative algorithms to reconstruct conductivity distributions with clear boundaries.^28^ Such solvers are easily accessible in open-source packages like PyEIT^3^ and EIDORS.^29^

In our present work, we hypothesize that a ML approach can provide superior reconstruction quality compared to these classic methods. This hypothesis is based on recent research that indicates that machine learning approaches, and more specifically neural networks such as Artificial Neural Networks (ANN), CNN and Deep Neural Networks (DNN), can effectively improve EIT reconstruction accuracy.^17,23,30^ In medical imaging more broadly, transformer-based models are another class of ML approaches that have demonstrated superior performance. They use attention mechanisms to capture long-range dependencies and global contextual information.^31,32^ However, ANN is limited in the number of network layers, which makes it easy for artifacts to appear in the reconstructed image. DNN training parameters are complex, and the training and image reconstruction processes are very slow, making it unsuitable for real-time image reconstruction.^17^ Similarly, the quadratic computational complexity of transformer attention mechanisms with respect to input sequence length creates substantial memory and processing overhead; additionally, they typically require large­(r) data sets and computational resources also in training.^33^

Meanwhile, CNN can preserve the spatial structure of data through operations like convolution and pooling,^34^ and thus remain attractive for EIT because they encode useful inductive biases (locality and translation equivariance), are parameter- and energy-efficient, and deliver faster inference suitable for real-time or embedded systems such as the one we are ultimately pursuing.^32^ Hybrid approaches, combining CNN with either attention and/or physics priors are likely to offer intriguing opportunities in the future, particularly as computing power continues to grow.

Given the advantages and our 1-dimensional measurement data, we build our ML reconstruction approach using 1D-CNN, relying on Python’s ML toolbox PyTorch (Figure S3; code freely available on GitHub^35^). As reference reconstruction algorithms we employ both EIDORS and PyEIT frameworks (GN and TV, respectively). We also employ EIDORS’ forward modeling to create synthetic, noisy data to train our ML reconstruction algorithm (see Table S3 for simulation parameters). Our resulting overall EIT validation framework is conceptually illustrated in Figure 2. In order to reduce the influence of outliers and to ensure a consistent reference scale across different samples and algorithms, we standardize conductivity element values by scaling to zero mean and unit variance within each image.

**Figure 2:**
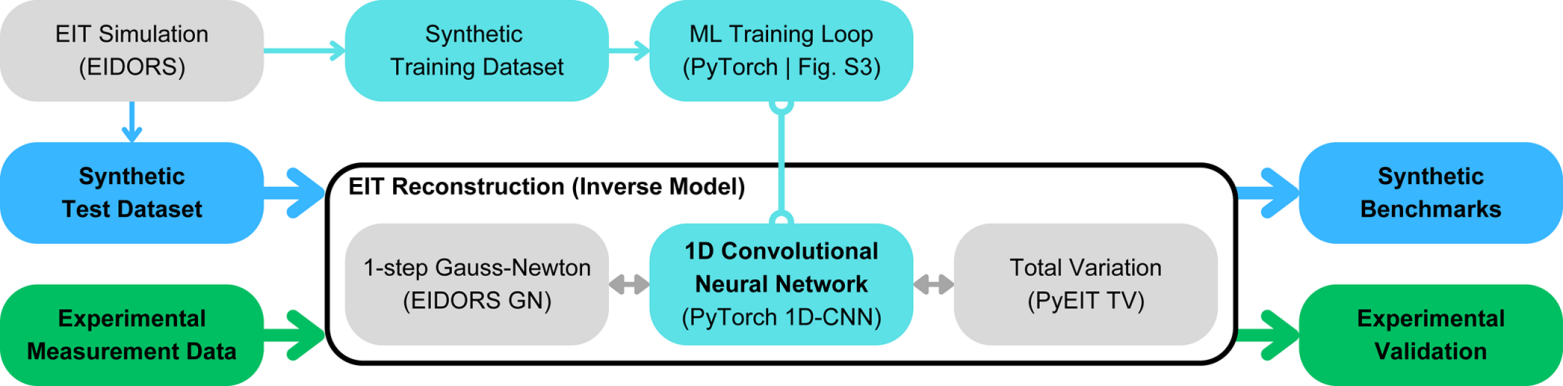
Framework for our EIT reconstruction. Synthetic data sets are first generated by EIT simulation software (EIDORS, based on Matlab) and loaded into the machine learning program built on PyTorch (Python ML toolbox; teal boxes/arrows). The 1D-CNN training involves multiple iterations and refinements (Figure S3) and is executed for different experimental parameter sets (Table S3). An independent test subset of the synthetic data (blue boxes/arrows) is then evaluated for reconstruction performance using our 1D-CNN and compared against one-step Gauss–Newton (EIDORS) and Total Variation (adapted for PyEIT) approaches. Finally, algorithm performance is validated across a range of experimental data (green boxes/arrows). All intermediate data processing (arrows) is implemented in Python.

## Results

Initially, we evaluate and compare the performance of different reconstruction approaches, namely GN, TV, and our 1D-CNN, on the synthetic test data set for both ad–ad and op–ad EIT excitation-measurement modes (section Synthetic benchmarks of EIT reconstruction). Subsequently, we evaluate EIT reconstruction performance using experimental data, ranging from various solid phantoms to biological entities (i.e., zebrafish egg and larva; section Experimental validation of EIT reconstruction).

### Synthetic Benchmarks of EIT Reconstruction

#### Qualitative Comparison of Reconstructed Images

Considering both the ad–ad and op–ad modes and independent of the amounts of objects, CNN appears to demonstrate consistent high fidelity (Figure 3). For single objects (shown in Figure 3A), the position, size, and contrast of the objects closely resemble the ground truth and there are very few reconstruction artifacts around the objects, including for the centrally located object, where sensitivity of either mode is lowest. This suggests that even the lowest sensitivity overall (ad–ad, array center) can still provide sufficient data quality for our CNN algorithm to effectively reconstruct. GN performs notably worse, and the excessive edge blurring seriously affects the estimation of sample size, although it can still provide a relatively accurate estimation of the sample position. In the images reconstructed using the TV method, the object can also be identified and their position closely resembles the position of the object in the ground truth. Object edges are better preserved than for GN, but for center objects is still notably worse than CNN. Additionally, TV appears more prone to generate small but noticeable artifacts (i.e., sizable regions of conductivity perceived as distinct from both object and medium, particularly near the electrodes in op–ad mode, see Figure 3aa,ab). These types of artifacts, also observed by, e.g., Zhou et al. are likely due to the high sensitivity near the electrodes^36^ and the so-called staircasing effect of the TV method itself.^37^

**Figure 3:**
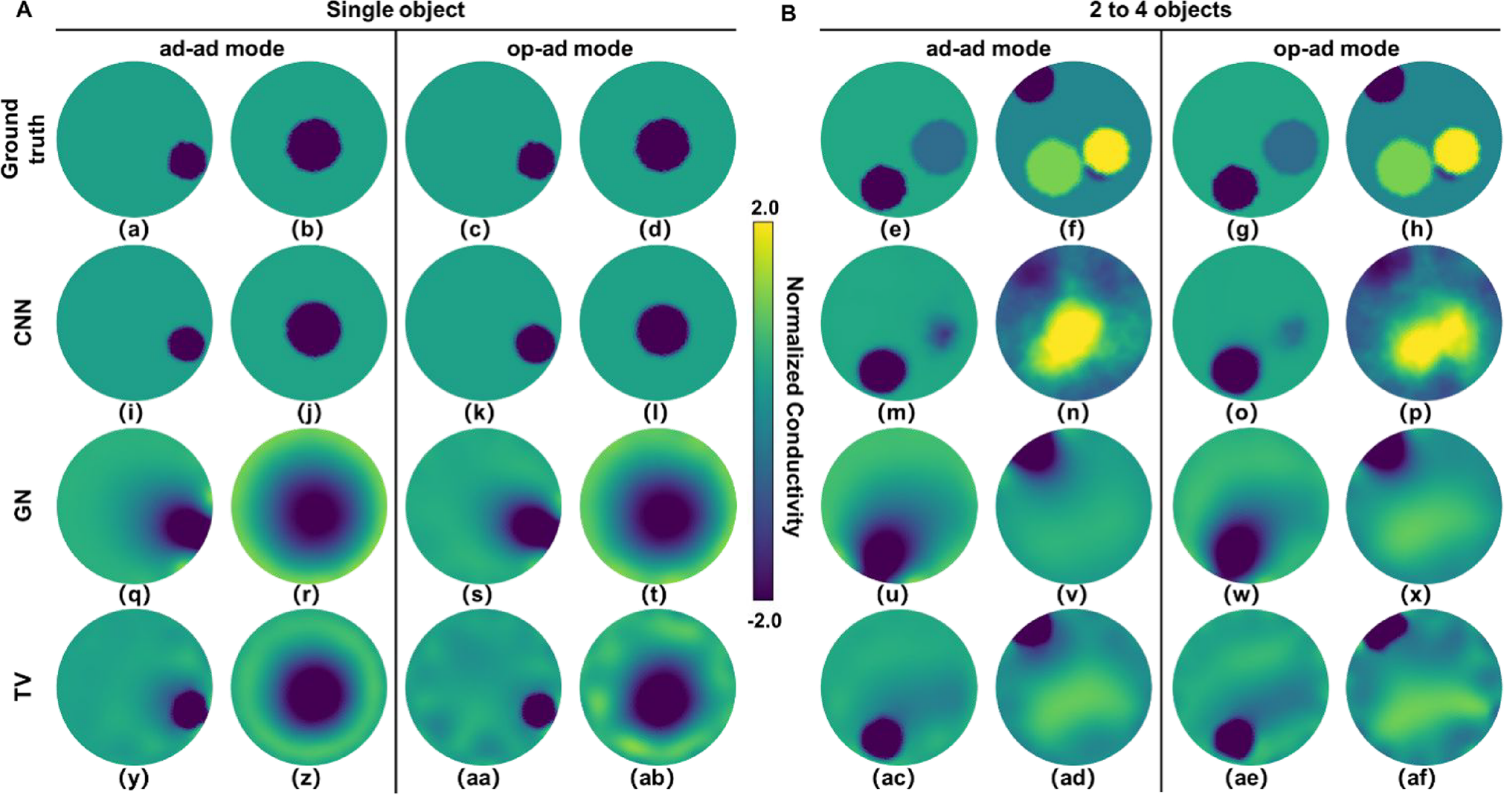
Reconstructed images of CNN, GN, and TV for different amount of objects in ad–ad and op–ad modes, alongside corresponding simulated inputs (ground truth). (A) Single objects: Regardless of whether the ad–ad or op–ad mode is used, CNN models can reconstruct images that closely match the object position and size of the ground truth. The reconstruction results of the GN method can provide position information, but extreme smoothing or blurring of object boundaries makes it difficult to estimate size. The TV method exhibits sharper boundaries compared to GN, though still less so than CNN, and appears to introduce artifacts near the electrodes (particularly in the op–ad mode, for example, as in image ab). (B) 2–4 objects: Showing representative examples with 2 and 4 objects. The reconstruction performance of the CNN models in both modes begins to decline compared to the single-object reconstructions, especially when the objects overlap, are small, or are close in conductivity (e.g., images n,p, middle 2 objects). In the GN reconstructions, the high-conductivity-contrast object at the electrodes appears blurred, similar to the single-object reconstruction, and dominant over the other objects that are only faintly present in the reconstructions. TV provides sharper boundaries for the same dominant object and captures conductivity distributions from other objects more clearly, but still with lower contrast than CNN. As with single-object imaging, artifacts near the electrodes are present. All methods provide superior contrast for centrally located objects under the op–ad mode (most pronounced with GN), highlighting the different sensitivity compared to the ad–ad mode.

Having multiple objects within the array significantly increases the computational complexity, as we allow the conductivity of each object to vary independently and also allow overlapping object positioning.^38^ Example reconstructions shown in Figure 3B reveal that CNN still allows for identification of object positions and sizes (especially in the op–ad mode), as well as some differentiation of conductivity differences (indicated by the color bar), though the ability of CNN models to clearly distinguish the boundaries of individual objects is reduced compared to the single object reconstruction. We also observe expected differences between the ad–ad and op–ad modes. For example, the low-conductivity-contrast object near the electrodes is more clearly reconstructed in ad–ad (Figure 3m,o). Conversely, the three more centrally located objects are effectively merged by ad–ad (Figure 3n), whereas op–ad provides clearer differentiation (Figure 3p), although conductivity differences are not properly displayed. Similar to the single-object GN reconstructions, multiobject reconstructions have large edge transition areas. Furthermore, the reconstruction results of the GN method are significantly affected by the position of the object, i.e. the ad–ad mode has higher sensitivity at the edge (Figure 3v, no central objects detected), and the op–ad mode has better overall sensitivity in the entire measurement zone (Figure 3x, central objects vaguely identified).^23^ The TV method continues to provide better object contrast than GN; however, it still exhibits clear deviations and omissions regarding the number and positions of objects compared to ground truth (Figure 3ac,ae). We posit that, like the artifacts that appear in the electrode area, this is because the TV method’s emphasis on preserving sharp boundaries necessarily encounters difficulties when both high-contrast and low-contrast objects are present (i.e., staircasing).^36,37^

#### Quantitative Evaluation of Performance

To evaluate algorithm performance quantitatively across more than a few selected reconstructions, we next compare 1000 CNN, GN, and TV reconstructions to the respective ground truth in terms of Mean Square Error (MSE). MSE is a commonly used metric for measuring the difference between two images. It calculates the square of the difference between corresponding mesh elements in the reconstructed and ground truth images and then averages them over all mesh elements, reflecting the average level of absolute error.^39^ The closer the value of MSE is to 0, the better the fidelity of the reconstructed image. In model evaluation, we further include both positive and negative controls to highlight the expected maximum range of the respective performance indicators. As positive control, we compare the ground truth image to itself. As negative control, we compare the ground truth image with a “random guess”, i.e., a randomly selected ground-truth image.

Performance quantification via MSE largely supports our qualitative conclusions, as CNN reconstructions yield the lowest median MSE values, showing a ∼4-fold improvement compared to GN and TV in the single-object case (Figure 4A). Though CNN performance degrades as the number of objects increases (Figure 4B; in line with reduced object contrast observed in Figure 3B; see also Figures S6 and S7), it maintains a clear advantage of at least 50%. Across all conditions, CNN also yields a noticeably narrower MSE distribution, i.e., fewer outliers, evident visually in the MSE distributions (Figure 4A), as well as in the ∼80% advantage in interquartile ranges (IQR, Figure 4B). Between excitation modes, MSE medians and IQRs with both CNN and TV are consistently in favor of the ad–ad pattern. We attribute this primarily to the fact that object positions are randomly generated in Cartesian (*x*/*y*) coordinates, meaning most objects are located near the electrodes rather than the center of the array,^40^ for which ad–ad naturally outperforms op–ad, as also observed qualitatively above (Figure 3A,B). Interestingly, with the GN method, the MSE under op–ad mode is marginally lower than that under the ad–ad mode. This may be because the GN, as the only linearized reconstruction method in our comparison, is more likely to suffer from the highly nonlinear sensitivity distribution in ad–ad mode, even with objects located near the electrodes, outweighing the trends observed in the nonlinear TV and CNN. This is reflected in images such as Figures 3v,x and S6, where edge-located objects in ad–ad mode dominate over ones located further toward the center of the array.

**Figure 4:**
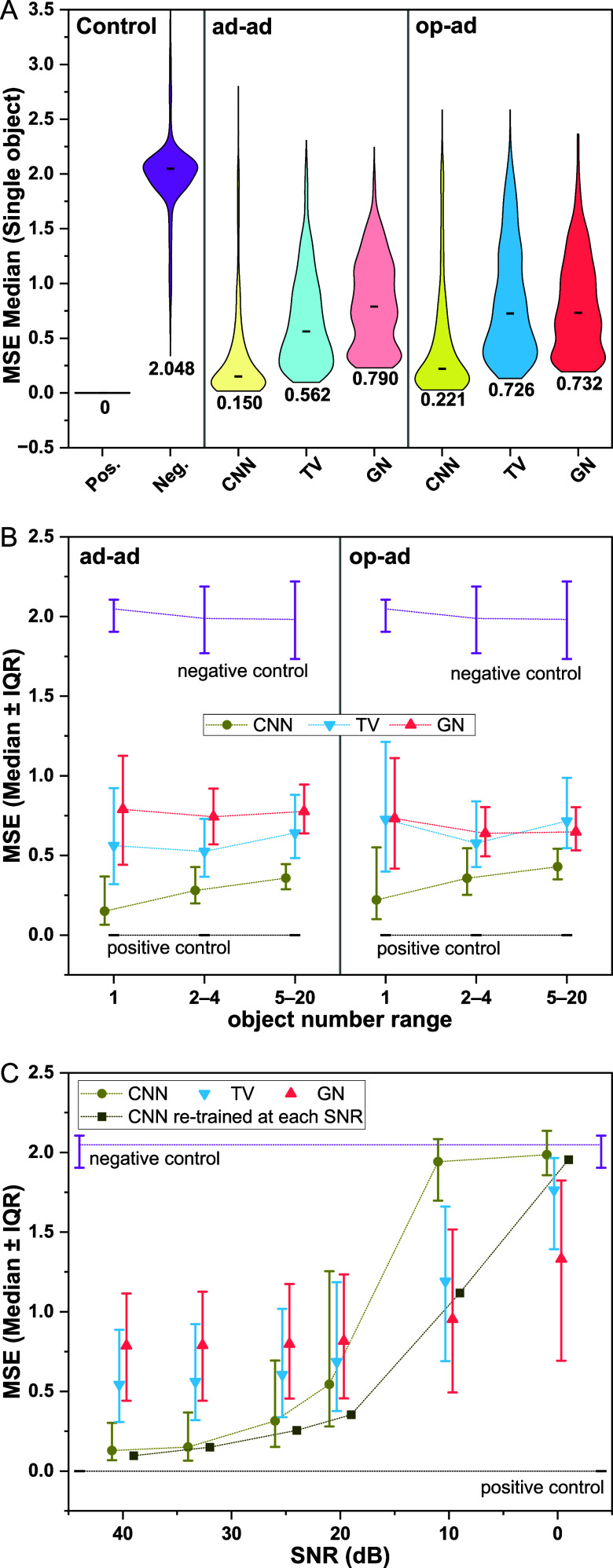
Quantitative performance of CNN, TV, and GN reconstruction. (A) Single objects: Violin plots showing the quantitative MSE comparison of reconstructed images (as well as controls, i.e., same ground truth and random ground truth) with ground truth across 1000 simulations from the test set. For both the ad–ad and op–ad modes, CNN performs closest to the positive control, whereas GN performs the worst, though still significantly better than the negative control. The TV method falls in between CNN and GN. In contrast to CNN and TV, which perform better in the ad–ad, GN yields smaller MSE under the op–ad mode (see text for discussion). (B) Multiobject comparison: MSE medians ± interquartile ranges (IQR) derived from distributions as in (A) for reconstructions of different object number ranges. CNN models yield the smallest errors and narrowest distributions across all conditions, followed by the TV and GN methods. The performance gap shrinks with the increasing object number, attributed in part to lower penalties for object smoothing and other artifacts, given a more complex ground truth (also reflected in wider negative control IQRs). (C) SNR comparison: MSE medians ± IQR as in (B), for single-object reconstruction in the ad–ad mode performed at different levels of synthetic SNR (data points are offset along the *x*-axis between methods for visual clarity only). The CNN model is robust within ±8 dB of its training SNR, maintaining a clear lead in MSE, but rapidly degrades below 20 dB. The other methods also show degrading performance with reduced SNR, the trend being weakest for the smooth GN method. CNN reconstruction performance at low SNR can be improved by retraining for the desired noise level (dark squares).

When considering trends for increasing object numbers in more detail, we interestingly find that, unlike CNN, MSE remains invariant or even decreases for TV and GN (Figures 4B, S5 and S6). To interpret this, it is relevant to note that at more than 1 object, a greater fraction of the chamber will exhibit conductivity different from the media, and – given our chosen property ranges – the object conductivities are likely to be correlated. This is also reflected in the increased IQR of the negative control MSE. At the same time, TV and GN are more likely than CNN to yield “blurred” object boundaries and similar artifacts around reconstructed object locations. In the multiobject case, even objects that are not “directly” reconstructed can still be covered by those boundary regions and thus in fact improve MSE (whereas the single-object case penalizes all such artifacts). This aligns with the fact that GN exhibits the largest boundary artifacts alongside the clearest trend of decreasing MSE median and IQR. With our CNN, the least likely to yield extended boundary artifacts, the opposing trend from increased reconstruction complexity dominates the median MSE – though the artifact-object-overlap phenomenon likely contributes to the narrowing IQR observed. TV, as expected, falls between these two extremes.

Finally, we take a closer look at the noise levels and robustness for single object reconstruction under ad–ad mode. The ad–ad mode is particularly considered as in this mode we observe the largest MSE variation between reconstruction methods. A synthetic SNR of 33 dB has been employed for CNN model training and all evaluation shown so far in order to match the lower end of real-life noise levels observed with our microEIT system (Figure S7). At higher SNR, the relative performance of all three reconstruction approaches is conserved, as seen in Figure 4C. Our CNN maintains a superior performance in terms of MSE also down to SNRs of 20 dB, with a sigmoidal jump however rendering it the worst reconstruction approach at SNR lower than 20 dB. This deterioration in performance at low SNR is not entirely unexpected – once we deviate far from the training data and training noise level, the CNN fails to distinguish relevant features from irrelevant ones (see also sample reconstructions in Figure S8). This is demonstrated by the dark squares, that show a CNN MSE recovery by retraining at a matching noise level. Conversely, GN benefits from its inherent smoothness and remains the most noise-invariant. TV shows an intermediate trend: at low SNR, its MSE is also higher than GN, which can be attributed to severe staircasing artifacts that arise under high-noise conditions.^37^ We expect that all reconstruction approaches could further benefit from hyperparameter retuning as the deviation from initial noise assumptions exceeds 10 dB (particularly for CNN, and to a lesser degree for TV).

### Experimental Validation of EIT Reconstruction

#### Object Conductivities, Quantities, and Positioning

While synthetic data is the only practical approach to train our CNN model, and synthetic benchmarks can provide a first valuable indication of its performance, it is ultimately real-world experimental performance that matters. For the evaluation of performance using experimental data, we limit our comparison to CNN and TV reconstruction, given that the GN algorithm produces overly smooth images. First, three ∼1.5 mm diameter objects made of different materials were used to test the image reconstruction capabilities of our CNN models for objects with various conductivities: a 3D-printed sphere (10^–11^–10^–21^ S/m), a tin ball (7–9 MS/m), and zebrafish eggs (chorion shell: 10^–7^ S/m; yolk core 0.2 S/m).^41^ The objects were placed in equivalent positions within the measurement chamber (i.e., similar radial distance from electrodes). Optical microscopy images and corresponding EIT reconstructions are shown side by side in Figure 5A. All methods correctly identify the conductivity gradients, i.e., that the tin ball is more conductive than the solution, and vice versa for the 3D-printed sphere and the zebrafish egg (dominated by the outer chorion). For all objects, CNN reconstruction produces sharper images compared to TV, and the reconstruction quality and observations align well with what we observed in the synthetic data.

**Figure 5:**
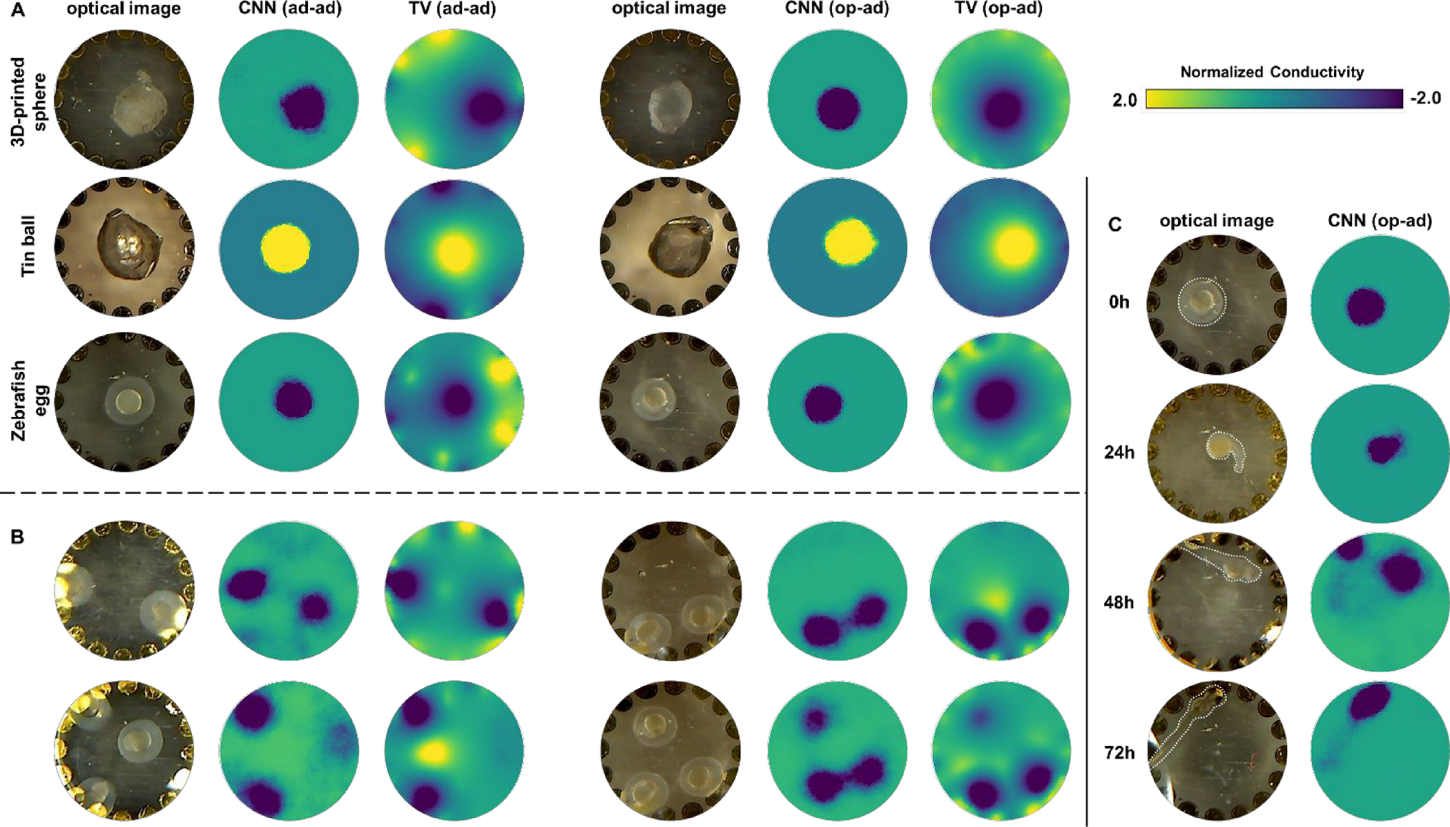
Microscopy images and reconstructed images of experimental objects. (A) Different materials. For all materials, the CNN models produce reconstructed images with sharper edges compared to the TV method in both ad–ad and op–ad modes, whereas both models result in relatively accurate size and positioning. (B) Different object numbers. When two eggs are present, our CNN models can accurately reconstruct both positions, with fewer artifacts compared to the TV method, in either mode. For three eggs, even the CNN model in the ad–ad mode was barely able to detect the more centrally located third egg. The op–ad mode shows a clear advantage in this scenario, particularly in the case of CNN reconstruction. To minimize the impact of lighting reflections (as retained for the higher-optical-contrast tin ball in A) on readability, we used software to remove some of the glare (the original images are available in Figure S9). (C) Zebrafish egg/embryo development monitored using op–ad EIT with CNN reconstruction. At 0 h, a fertilized zebrafish egg was imaged. At 24 h, the chorion of the zebrafish embryo was removed to better observe the embryonic morphology, which resulted in reduced contrast in the EIT images reconstructed at 24 and 48 h. The reconstructed images at 48 and 72 h reveal the shapes of the zebrafish larva, although all of the objects in the training data set were circular. This indicates that our CNN model can, to some extent, still distinguish the shapes of the zebrafish.

It moreover appears that size estimation is more reliable with CNN than with TV, particularly in ad–ad mode, which again matches our synthetic-data observations. However, the tin ball size appears to be underestimated even by CNN. We expect that this is due to the conductivity of the tin ball being well beyond the training conductivity range. A consistent underestimation for our CNN approach could have been expected as synthetic training data considered only in-plane conductivity, whereas our experiments deal with spheres located just above the electrode plane. To evaluate this, we performed experiments with 3D-printed cylinders of varying height placed at varying vertical separation from the electrode plane. Figure S10 shows that this effect is minimal at ∼5% for <1 mm out-of-plane, similar to the spheres shown in Figure 5A.

Our ultimate goal lies in applying EIT to biological entities with inherently lower conductivity contrast (for which our CNN was primarily trained), thus, we proceed with further evaluation of object accuracy for zebrafish eggs only. To quantitatively evaluate how well our CNN models perform in reconstructing the egg positions and dimensions, we apply image segmentation to both optical images and corresponding CNN EIT reconstructions (Figure S11). Our analysis yielded small average positional errors of ∥147 μm∥ (∥4%∥) in zebrafish position and +70 μm (+6%) in their diameter. Positional errors are distinctly larger for eggs near the edge of the array, attributed to the optical image distortion (refraction) from the increased liquid meniscus curvature near the chamber’s edge. We can further attribute part of the sizing error to image segmentation. Overall, op–ad mode appears to be more reliable on position estimation throughout the chamber. We further observe differences in size estimation accuracy between op–ad and ad–ad modes that align with our expectations from respective sensitivity distributions, as discussed above.

In Figure 5B, we further evaluate the reconstruction performance of our CNN models (ad–ad pattern and op–ad pattern) and the TV model, this time with multiple zebrafish eggs in the chamber. For two eggs, both excitation/measurement patterns can still achieve good image reconstruction results using CNN models, with less imaging artifacts and sharper edges compared to the TV model. Especially in the image reconstruction of the CNN model with op–ad pattern, even when the two eggs are close to each other, the reconstructed image still shows the edges of both eggs. Most notably, with three eggs in the chamber we can begin to demonstrate the benefits of the op–ad mode with its higher sensitivity in the central area.^23^ On the contrary, the ad-ad mode can only provide an indication of a centrally located egg, as the large voltage differences from the eggs nearer to the electrodes lead to the signal from the middle one being filtered out as noise. The TV algorithm does not allow for reconstruction of the centrally placed egg. In op–ad mode, however, all three eggs are clearly reconstructed by CNN, and even TV gives at least a vague indication of a third object. This experiment also showcases the reduced sensitivity of op–ad mode at the electrodes, with the two-egg reconstructions showing lower contrast compared to ad–ad mode with either algorithm, though both eggs could still be resolved. We attribute the ad–ad/op–ad differences apparent here to the inherent low conductivity contrast between medium and objects, compared to a wider range being present in the synthetic data.

#### Zebrafish Embryo Development Process

From the above tests, we have established that the CNN models using the ad–ad and op–ad patterns have stronger image reconstruction capabilities compared to the TV model. Among them, the op–ad model shows better real-world performance in terms of overall sensitivity in the whole measurement zone. We thus proceed to a final, more application-focused demonstration of its capabilities, where we use our CNN-based EIT reconstructions to capture the morphological evolution of a zebrafish embryo over a 72-h period. As shown in Figure 5C, the reconstruction correlates visually with the optical images, though showing reduced visual fidelity compared to the simple egg shape. We attribute this reduced fidelity to two factors. First, the nature of our CNN training data, specifically the training on 2–4 circular objects, leads to reconstruction bias, seen, for example, at 48 h where the fish larva is reconstructed as two circles. At 24 and 72 h, actual larval shapes are well approximated as overlapped circular objects. Overall, the model still captures general developmental trends, demonstrating robustness even without task-specific training. Further refinement with a dedicated, more targeted CNN trained on single-ellipse-objects showed some benefit for the later time points (Figure S12). Early time points, however, correspondingly suffer, pointing toward trade-offs in model generalization. Second, image contrast for the 24-h (midto-late segmentation period) and 48-h (late pharyngula/early hatching period) time points is lower than either the embryo or larval stages. This in fact aligns well with the underlying biological transitions. In these intermediate time points, the high-resistivity outer chorion layer of the embryo has been removed, but the larva’s skin structure remains immature, consisting primarily of a thin basal layer and an outer layer known as the periderm. By 72 h, the skin structure becomes fully developed, with a clearer differentiation and hierarchical organization of the epidermal cells.^42^ This increased protective function also gives rise to an increased resistivity, and thus better EIT contrast.

## Conclusions

In this study, we demonstrate, for the first time, a micro-EIT platform with a glass-based electrode chip (4 mm diameter measuring zone) as the core component, offering modularity, biocompatibility, and transparency. Alongside the EIT chip, we introduced custom Python-based 1D-CNN models for image reconstruction, which we trained and validated using synthetic and experimental data. These models achieved sharper object boundaries, more accurate object counts, and reduced artifacts compared to traditional approaches such as one-step Gauss–Newton and Total Variation methods. By open-sourcing our 1D-CNN, we provide a free high-performance alternative to existing reconstruction tools for micro-EIT systems.

Our 1D-CNN models were specifically built, optimized, and trained using a parameter range in line with biological EIT application, and corresponding to different numbers of objects and different excitation-measurement modes. For initial benchmarks, we employed synthetic data generated using the EIDORS toolkit (analogous to, but independent of, the training data) in order to compare CNN models against GN and TV both qualitatively and quantitatively. First, synthetic data was loaded into our ML models, as well as the traditional GN and TV models for comparison. Under both ad-ad and op–ad EIT excitation-measurement modes, our CNN models offered >3× lower reconstruction image error than the other methods when a single object was present. Performance was reduced for increasing object numbers, but with CNN still maintaining a ∼2× lower MSE compared to GN and TV.

During experimental data testing, our CNN models successfully reconstructed images of objects composed of different materials covering a conductivity range of >20 orders of magnitude. CNN reconstruction was worst for the tin ball, whose conductivity was well beyond the training-set range, resulting in size underestimation; however, positional accuracy remained good. The CNN showed consistently better reconstruction quality, with sharper contrast and lower artifacts, compared to TV across all experimental tests. We moreover quantified the positional and dimensional accuracy of images reconstructed using our CNN models and found that average errors relative to optical imaging were around 5%. In a multiple-object test, we further confirmed the advantage of using the op–ad excitation/measurement mode, for imaging objects in both the center and the edges of the measurement area.

Finally, we showcased a practical use case of our micro-EIT system by monitoring zebrafish development from embryo to larva over 72 h. Therein, we could clearly observe changes in shape and conductivity using CNN reconstruction in line with the underlying biological development. To the best of our knowledge, this study represents the first attempt to image the developmental process of zebrafish embryos using EIT, a particularly challenging reconstruction task given that the shape of the object changes during the course of observation, another fact that speaks to the robustness of our CNN models.

Our micro-EIT platform and chip have thus proved robust and amenable to both optical observation and EIT measurement. Our gold-on-glass approach could, in the future, be improved further by implementing indium–tin oxide for a fully transparent device. Another clear goal lies in further downscaling of the electrode array, which will necessitate further investigation of how this may affect model performance. Although our CNN models demonstrated significant advantages in imaging performance, a key remaining limitation lies in the overly simplistic synthetic training data (smooth convex objects, homogeneous permittivity and resistivity). Along with the low measurement frequency employed, this leads to a lack of resolving power for the more complex internal and external structure of biological objects (from cells to zebrafish). In the future, we intend to further explore how to balance broad model applicability (e.g., across zebrafish developmental stages) with more narrowly application-tailored training set parameters, alongside multifrequency EIT measurements. Given the favorable performance of our physical system and computational framework, we expect that micro-EIT can be developed into a reliable complementary tool for observing biological processes.

## Supplementary Material



## Data Availability

Our code is freely available on GitHub.^35^ The 1000 synthetic simulations and reconstructions are freely available at LeoPARD.^43^

## Notes

Detailed information on chip fabrication, measurement setup, and CNN training; quantification for higher object numbers and for single-object position/size; and original optical images (PDF)

## Abbreviations

EIT: electrical impedance tomography
1D-CNN: 1-dimensional convolutional neural networks
ML: machine learning
ad-ad: adjacent injection–adjacent measurement pattern
op–ad: opposite injection–adjacent measurement pattern
TV: total variation method
GN: one-step Gauss–Newton method
ANN: artificial neural networks
DNN: deep neural networks
MSE: mean square error
