# Supplementary material for: A Comparative Evaluation of Microimpedance Tomography Reconstruction Algorithms for in Vitro Imaging
Source: ACS Sens. 2025 Nov 6;10(11):9093–102. doi: 10.1021/acssensors.5c03758 (PMC12670993; doi:10.1021/acssensors.5c03758)
Supplement: Supplementary file 1 [file se5c03758_si_001.pdf]

Supporting Information: A Comparative Evaluation of Microimpedance Tomography Reconstruction Algorithms for in Vitro Imaging

Chang Liu,[†,ǁ] *Xingyang Chen,*[†] *Thomas E. Winkler,*[*,†,‡,§] *and Iordania Constantinou*[*,†,ǁ]

[†]Institute of Microtechnology (IMT), Technische Universität Braunschweig, Alte Salzdahlumer Straße 203, 38124 Braunschweig, Germany

[‡]Micro and Nanosystems (MST), KTH Royal Institute of Technology, Malvinas väg 10, 100 44 Stockholm, Sweden

[§]Digital Futures, KTH Royal Institute of Technology, Malvinas väg 10, 100 44 Stockholm, Sweden

[ǁ]Center of Pharmaceutical Engineering (PVZ), Technische Universität Braunschweig, Franz-Liszt-Str. 35a, 38106 Braunschweig, Germany

*Corresponding author e-mail: winklert@kth.se, i.constantinou@tu-braunschweig.de

Table S1. Micro-EIT chip fabrication process.

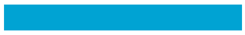	**Wafer clean** (0.7 mm 4-inch glass wafer)<br>• Piranha (optional)<br>• Quick Dump Rinser (QDR) to wash the wafer with deionized (DI) water<br>• Acetone 1 min<br>• Ethanol 1 min<br>• Baking at 110°C for 2 min
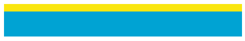	**Sputtering**<br>• High pressure air blow, remove particles<br>• Sputtering machine: Ti 10 nm, Au 300 nm
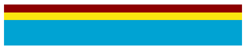	**Spin coating photoresist**<br>• Hexamethyldisilazane (HMDS) treatment, at 115 °C, 5 min<br>• 1.25 ml ma-P 1215 (micro resist technology GmbH) 3000 rpm, 30 s<br>• Prebaking on hotplate, at 110 °C, 2 min
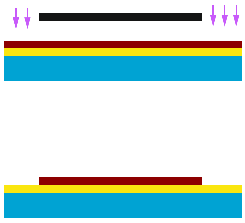	**Exposure**<br>• Mask aligner, top exposure mode<br>• Mask for electrodes and wires (part of the electrodes and wires are covered)<br>• Align the positions of mask and wafer<br>• Recipe: Dose 25 mJ/cm^2, Separation 30 μm<br>• Development: wafer put into ma-D 331 (micro resist technology GmbH), keep shaking the wafer, until large exposed area without photoresist (min. 30 s)<br>• Check under microscope with yellow light filter to see if there are bubbles under photoresist<br>• After baking on hotplate, 110 °C, 5 min
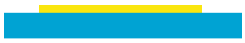	**Au etching**<br>• O_2 Plasma treatment (O_2 20 sccm, 100 W, 200 mTorr) for 5 min<br>• Au etching solution (Iodine : Potassium iodide : Water = 1 g : 2 g : 20 ml), keep shaking the wafer for around 1 min. Wafer can be washed time by time to check if all exposed area without Au<br>• DI water washing, until no Au etching solution on wafer surface<br>• Wash wafer in first Acetone container for 1 min, then in second Acetone container for 10 s, then in Ethanol container for 10 s, to remove photoresist

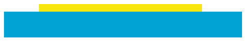	**Ti etching**<br>• Put wafer in Ti etching solution (320 ml DI water, 120 ml 65% HNO_3, 25 ml 40% HF), shaking around 10 s, etched part should be totally transparent<br>• Use QDR to wash the wafer for 5 min<br>• Baking on hotplate, 110 °C, 2 min<br>• Barrel Etcher: O_2 Plasma (O_2 20 sccm, 100 W, 200 mTorr)
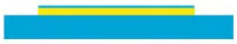<br>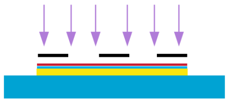<br>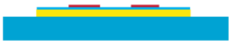	**SU-8 5 (as protection layer)**<br>• Two hotplates preheating at 65 °C and 95 °C<br>• Load wafer on spin coating machine, use dispenser to apply 5 mL SU-8 5 on wafer, speed at 1400 rpm<br>• Prebaking on hotplate, 65 °C 2 min, 95 °C 6 min<br>• Clean SU8 spin coating machine<br>• Mask aligner, using mask for electrode holes (electrode parts are covered), top exposure mode, set Dose 180 mJ/cm^2, Separation 30 µm<br>• Baking before development on hotplate, cover the wafer with a petri dish, 65 °C 1 min, 95 °C 1.5 min, cooling down. Adjust hotplate to 160 °C<br>• Development: PGMEA (Propylene Glycol Monomethyl Ether Acetate) development solution 2 min, cleaning solution (PGMEA) 10 s, Isopropanol 10 s, keeping shaking. Spin dry<br>• After baking on hotplate, 160 °C 10 min (or even longer)<br>• Check connection between small and large electrodes
	**O2 plasma treatment to remove photoresist residue**<br>• Barrel etcher, O_2 50 sccm, 200W, 200 mTorr, 2 min

Table S2. Relevant properties of 0.3× Danieau solution.

Property	Value
Conductivity	0.16–0.19 S/m
NaCl	17.4 mM
HEPES	1.5 mM
KCl	0.21 mM
$Ca(NO_3)_2$	0.18 mM
$MgSO_4$	0.12 mM

Table S3: Key parameters employed in our forward modeling framework.

We rely on synthetic datasets generated by EIDORS as it allows for flexible adjustment of EIT simulation parameters, such as conductivity, measurement noise, object position/number/size, and electrode configurations, thereby producing diverse training data.[1] Parameters were set in line with our experimental setup and with a wide range of biological environments and objects. We omitted out-of-phase contributions from the simulation, since we expect the impedance contrast between objects and medium to be dominated by in-phase resistance/conductivity at the selected frequency.[2] Each synthetic dataset had 100,000 simulations split into two subsets (**Figure 2**): 80,000 simulations in the training set for ML model training (see **Figure S3**; including a validation sub-set of 16,000 for model evaluation during the training loop),[3] and 20,000 simulations as a test set used to assess the performance of the final model (i.e., the basis for all our analysis in the present paper). Each simulation includes – both for the objects, and the matching empty-chamber reference – a conductivity map, as well as an array of idealized electrode-pair voltages $U_{i,j}$ (plus corresponding $V_{i,j} = U_{i,j} + rnd(\text{SNR})$, i.e., with added normally-distributed noise according to the desired SNR).

Parameters	Value(s)
Injected current	0.1 μA
Injection mode	ad-ad *or* op-ad
Chamber diameter	1 mm [a]
FEM refinement	0.02 mm (4684 elements)
Medium conductivity	0.1–2 S/m [4]
Object number	1 *or* 2–4 *or* 5–20
Object diameter	0.1 to 0.4 mm [a]
Object conductivity	10^{-5} to 1 S/m [5]
SNR	33 dB [b]

[a)] The chamber size selected for EIDORS was a unit size, which was used for investigating the possibility of being applied to a range of micro-scale EIT chambers (~0.5–5 mm, i.e., including our present 4 mm chamber). The object diameter range equates 10%–40% relative to chamber diameter, matching the objects in our later experiments.

[b)] see **Figure S7** for details on the noise assessment for our experimental setup

Table S4: Key (hyper)parameters used in our reconstruction algorithms.

As illustrated in **Figure 2**, we employed and compared three EIT reconstruction methods for our synthetic and experimental data (see **Figure S2**): one-step GN, alternating direction method of multipliers TV, and our trained 1D-CNN model (see also **Figure S3**). All reconstruction methods include a range of (hyper)parameters that were empirically tuned as shown below.

**Method**	**(Hyper)Parameter**	**Value**
GN	Regularization weight (λ)	10^{-5}
TV	Regularization weight (λ)	10^{-4}
	Penalty term (ρ)	1.5
	Max iterations	50
CNN	Learning rate	0.001 [a]
	Epoch	100 [b]
	Batch size	256 [c]

[a)] The learning rate is a parameter used to adjust the speed at which a model learns. If the learning rate is too high, the model may fail to converge, while a rate that is too low can lead to slow training or getting stuck in a local optimum. Typically, the learning rate is selected to be in the range of 0.001 to 0.01. To prevent model divergence, we chose 0.001.[6]

[b)] Epoch refers to the number of times the model is trained on the same dataset during the training process and can lead to underfitting (too few) or overfitting (too many).[6] For our empirical evaluation results see **Figure S4.**

[c)] Finally, increasing batch size speeds up the training process and makes it more stable, but at the same time more likely to get stuck in a local optimum. A smaller batch size, while making the training process less stable, is more likely to achieve a global optimum.[7] For our empirical evaluation results see **Figure S4.**

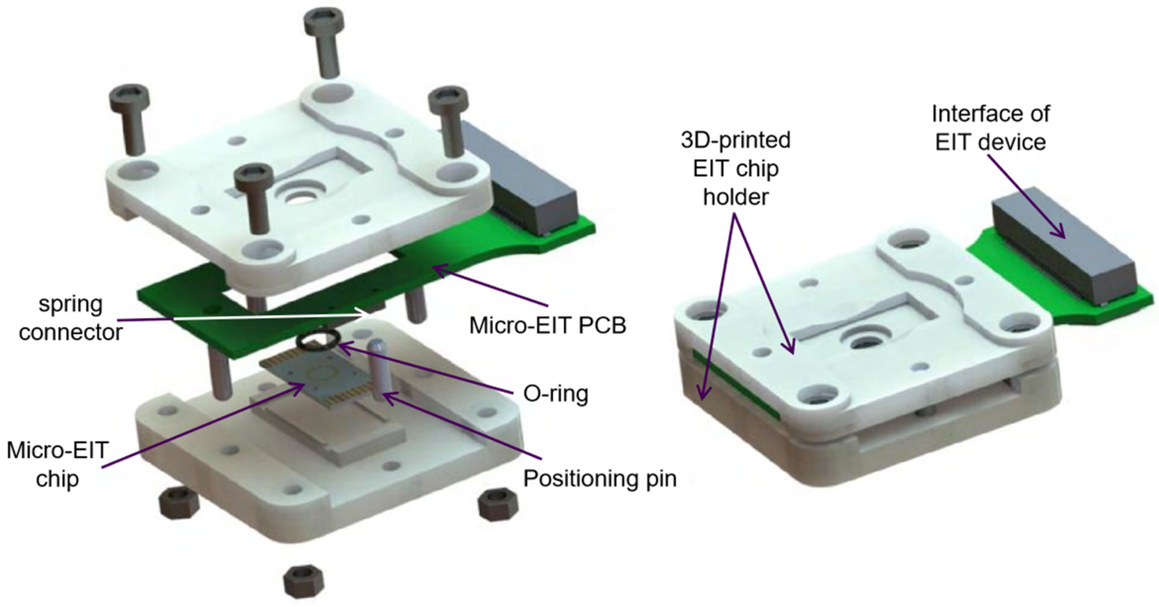

Figure S1. 3D schematic illustrating our 3D-printed assembly holding the glass micro-EIT chip at its core, with a PCB connecting the chip to the measurement instrumentation, specifically a Sciospec EIT32 platform. To relay measurement outputs to our custom image reconstruction framework (discussed in paper section EIT image reconstruction), we build a custom Python-based graphical user interface that integrates instrument control (e.g., current amplitude and frequency, excitation pattern), reconstruction control (algorithm, channel to electrode mapping), and associated data input/output (code made freely available on GitHub).[8] Optical top-view images of the measurement chamber are captured to serve as a comparison to the reconstructed electrical images using a TOOLCRAFT BN 1713197 micro-camera. Using our GUI, 1 frame/s real-time EIT reconstructed images and corresponding optical micrographs can thus be observed concurrently.

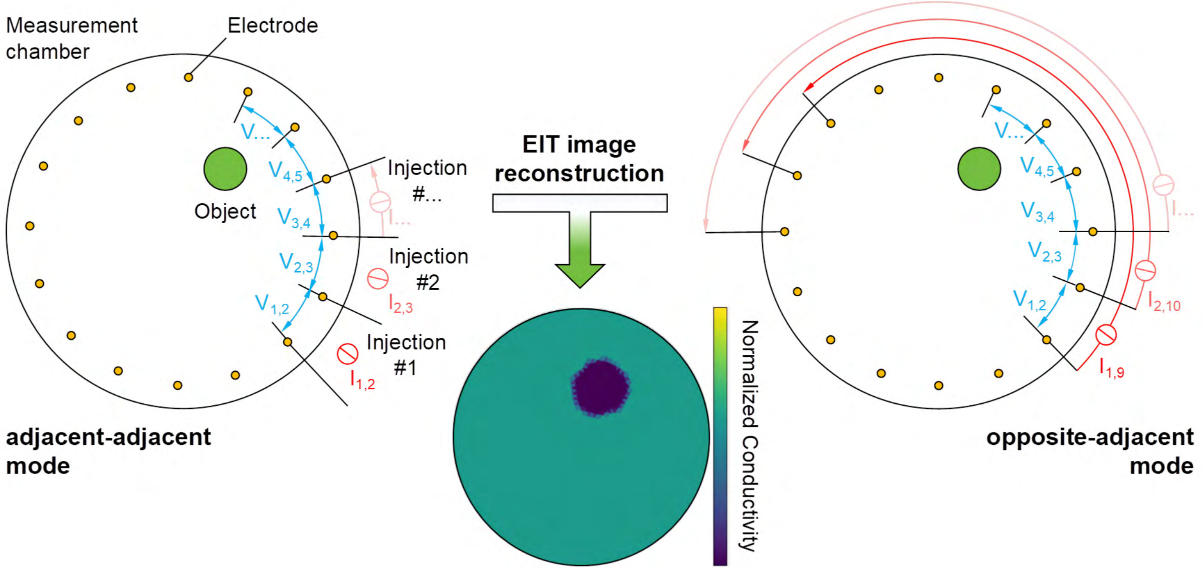

Figure S2. Principle of EIT measurement (and forward modeling). Adjacent (injection) - adjacent (measurement) pattern (ad-ad; left) and opposite (injection) - adjacent (measurement) pattern (op-ad; right) are illustrated in a chamber with 16 electrodes arranged in a ring configuration. A representative circular object is shown in the measurement chamber. Red arrows indicate the pairs of electrodes used for current injection and blue arrows indicate the pairs of electrodes used for voltage measurement. Current is injected between two electrodes at a time according to the chosen measurement pattern, and the voltage is measured between all 16 adjacent-electrode pairs. A ms-scale delay between injection and measurement allows the electric field to stabilize within the imaging chamber. With all 16 possible injection pairs, a complete EIT image thus relies on 256 measurements for the op-ad measurement pattern and 208 for the ad-ad pattern, as we exclude measurements that include the injection electrodes to avoid the confounding impact of the surface impedance.[9,10] Additionally, all our experiments include an initial set of reference measurements of an "empty" (i.e., solution-only) chamber. All analysis is based on accordingly normalized voltages (and, more specifically, the real in-phase voltages), i.e., $\left(Re(V_{i,j}^{\mathrm{obj}}) - \mathrm{Re}(V_{i,j}^{\mathrm{ref}})\right)/\mathrm{Re}(V_{i,j}^{\mathrm{ref}})$. Forward modeling in EIDORS follows an analogous process, with parameters shown in **Table S3**. These measurements or forward-model simulations provide the basis for all EIT image reconstruction (bottom/middle).

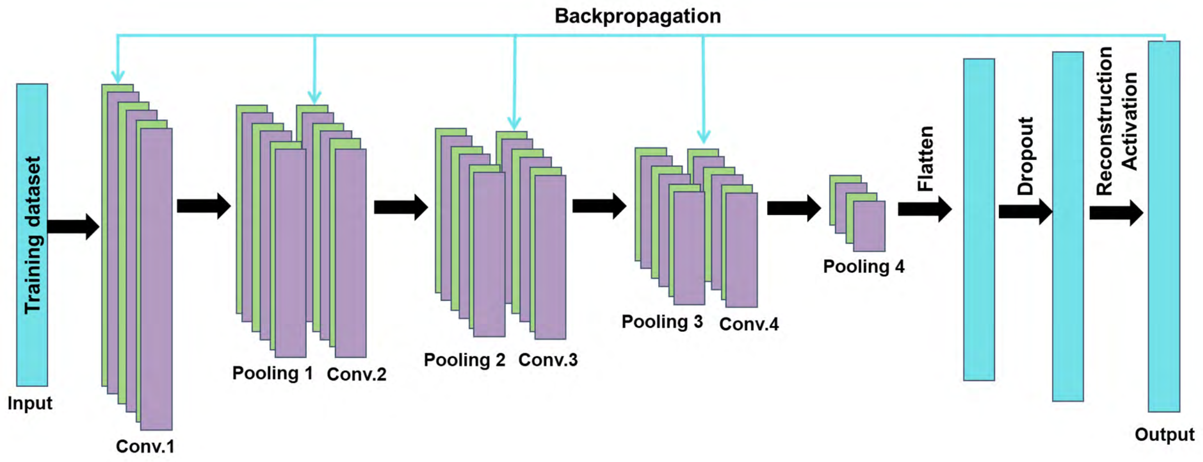

Figure S3. **CNN Training loop.** The training dataset is loaded into PyTorch for ML model training (see **Figure S2** and **Table S4**). To optimize the performance of our 1D-CNN, four convolutional layers were used to extract data features and recognize data patterns. This configuration was chosen as it represents the minimum number of layers sufficient to accomplish the task, helping to reduce computational cost while maintaining performance. Simultaneously, four pooling layers were used to downsample and compress the extracted features, reducing computational cost and enhancing the model's robustness to noise.[6] Afterwards, a full connected layer and dropout are used to prevent overfitting.[11] At the end of each training epoch, the output results undergo backpropagation to update the parameters and gradients of the neural network, followed by the next round of training.[6] In the process of training CNN models, there are several key hyperparameters that need to be adjusted: learning rate, epoch, and batch size, which have significant effects on the outcome of machine learning. These are summarized in **Table S3**, with empirical evaluation in **Figure S4**.

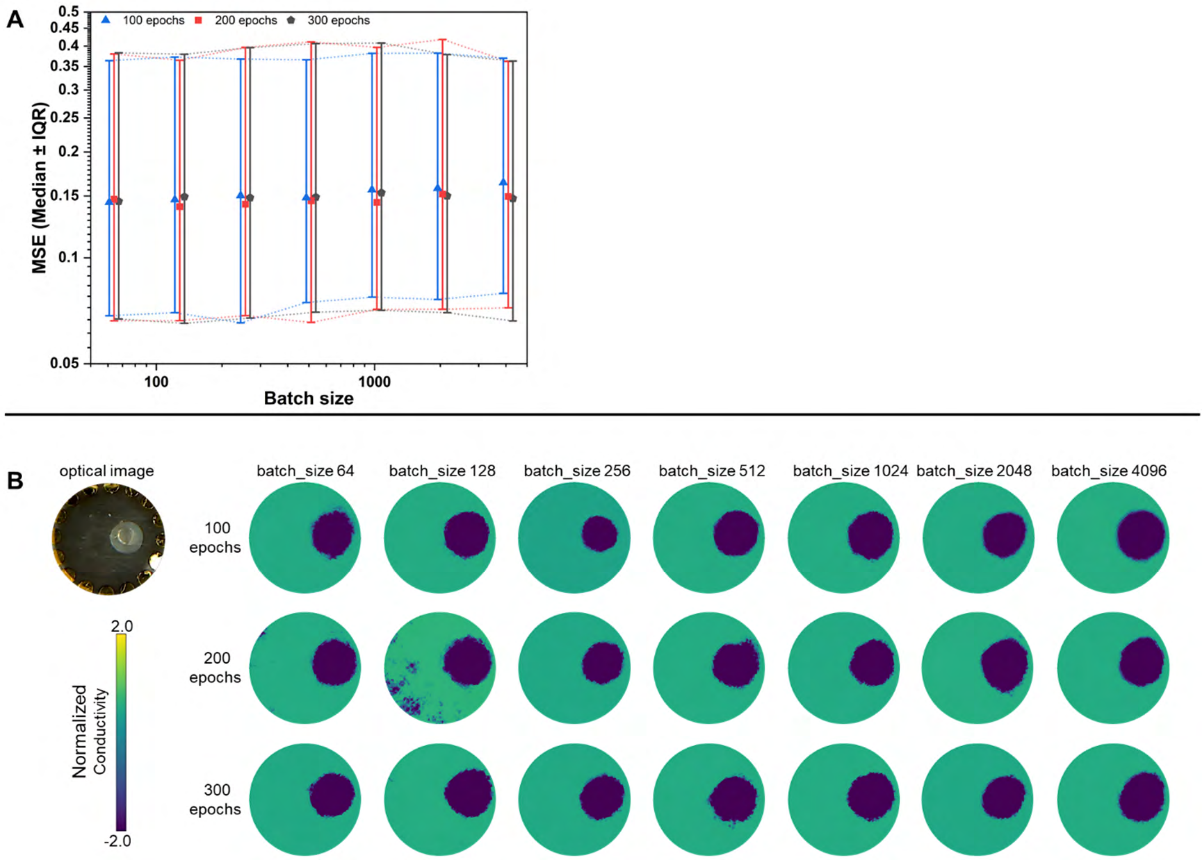

Figure S4. ML hyperparameter selection. (A) The effect of batch size and epoch number on the mean squared error (MSE) during model training. Variation in medians and IQR is overall minimal. For shorter training (100 epochs), we observe the clearest trend regarding batch size, with medians generally increasing with batch size, and Q1 following a more V-type pattern. This would be in line with mild overfitting at smaller batch sizes, while excessively large batch sizes may drive the optimizer toward sharp local minima, resulting in suboptimal convergence and degraded generalization performance.[12] Prolonged training (200-300 epochs) flattens out the trend and generally increases Q3, which could be an indicator of overfitting. With a combination of lowest Q1 and low Q3, 100 epochs and batch size 256 appears optimal.

(B) Because under- and overfitting cannot be conclusively assessed only using purely synthetic data, we further examined experimental EIT reconstructions, with one representative zebrafish egg example shown here. The best size correspondence is consistently obtained for 100 epochs and batch size 256, in agreement with the MSE evaluation. Other hyperparameter combinations yield oversized reconstructions and occasional clear artifacts, indicating under- and/or overfitting.

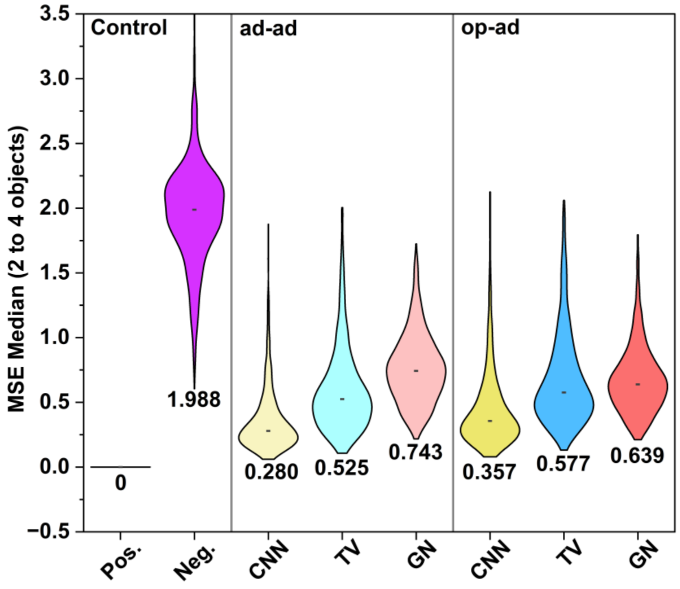

Figure S5. Performance of CNN, TV, and GN reconstruction in ad-ad and op-ad mode for 2–4 objects. Violin plots showing the quantitative MSE comparison of reconstructed images (as well as controls, i.e., same ground truth, and random ground truth) with ground truth across 1,000 simulations from the test set. The distributions underly the summary statistics shown in **Figure 4B**; corresponding sample reconstructions are shown in **Figure 3B**; for associated discussion, we refer to the manuscript.

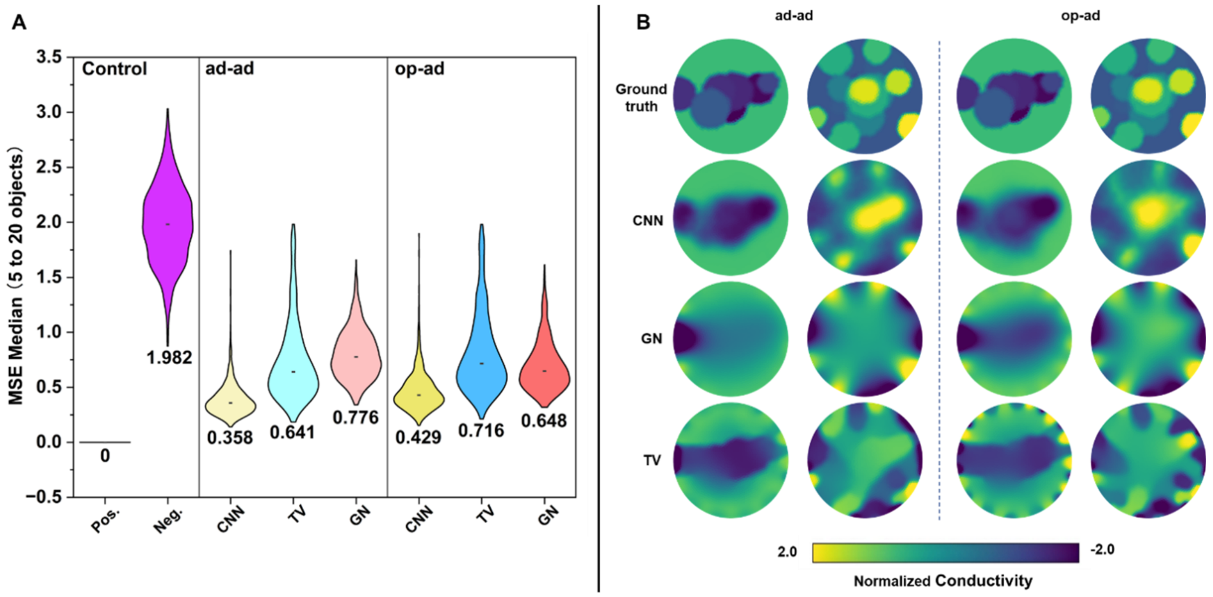

Figure S6. Performance of CNN, TV, and GN reconstruction for 5 to 20 objects in ad-ad and op-ad mode. (A) Violin plots showing the quantitative MSE comparison of reconstructed images with ground truth across 1000 simulations from the test set of 5 to 20 objects. The distributions underly the summary statistics shown in **Figure 4B**, and are discussed in the main text. **(B)** Image reconstruction results for 5 to 20 objects. Similar to single-object and 2-to-4-object benchmarks, under both modes, the reconstruction results of the CNN models yield the clearest correspondence, and GN the greatest loss of detail.

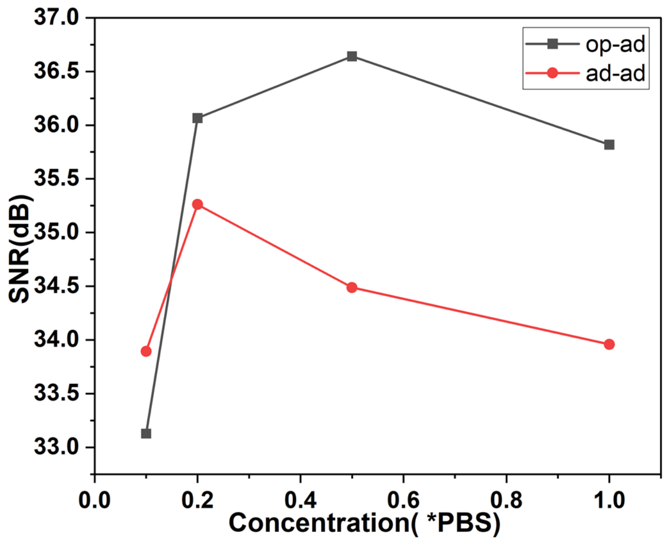

Figure S7. Experimental SNR. We performed EIT measurements on PBS solutions of different concentrations (0.1×, 0.2×, 0.5× and 1×; corresponding approximately to a conductivity range from 0.16 to 1.6 S/m) to determine noise levels in our setup, and to what extent they were dependent on medium conductivity. For each concentration, 20 frames of voltage data were collected, from which we calculated the mean absolute voltage $\overline{|V|}$ as well as standard deviation $\sigma(V)$ from the mean array to derive $\mathrm{SNR}_{dB} = 20 \cdot \log_{10}\left(\overline{|V|}/\sigma(V)\right)$. As shown in the figure, the SNR did not follow a clear trend with conductivity, and consistently tested above 33 dB, which was thus chosen as the baseline SNR for synthetic dataset generation.

As the measurements were conducted in a low-noise laboratory environment, and given the relatively high measured SNR and the low excitation current (1 μA) and frequency (10 kHz) used, no additional electromagnetic shielding was implemented, as it was found unnecessary for achieving stable and reproducible results.

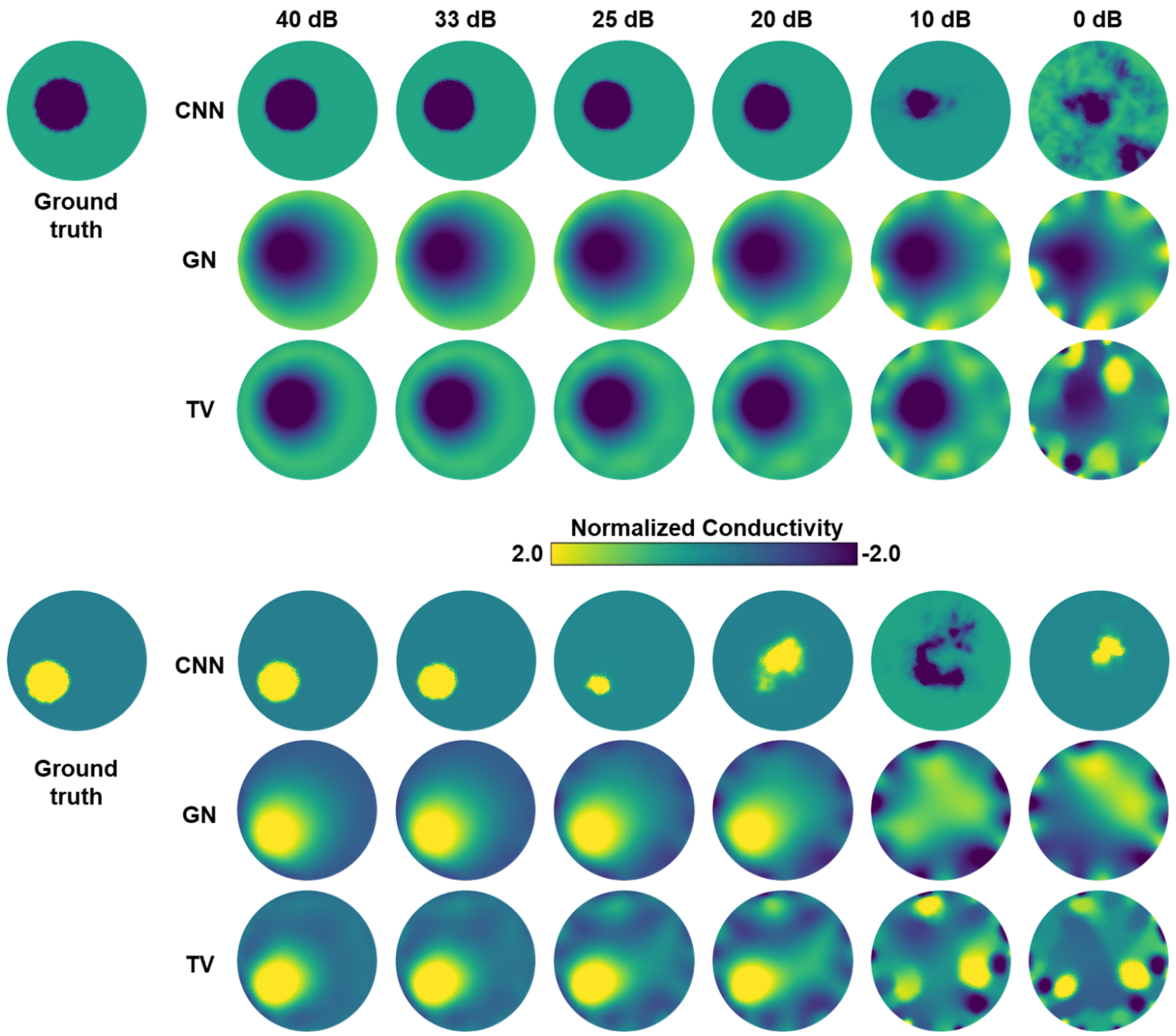

Figure S8. EIT reconstruction performance at different SNR. Two sample reconstructions using CNN, TV, and GN for single-object synthetic data in ad-ad measurement-excitation mode under a range of synthetic SNR scenarios (see **Figure 4C** for corresponding quantitative analysis). Our CNN was trained on data with 33 dB SNR, corresponding to experimental conditions (**Figure S7**). While stable at higher SNR, CNN performance degrades towards as conditions deviate substantially (>10 dB) from the training dataset, with artifacts (top sample, 0 dB) and hallucination (bottom sample, 20 dB). The overly-smooth GN reconstruction proves the most stable, with TV yielding intermediate results, as discussed further in the manuscript.

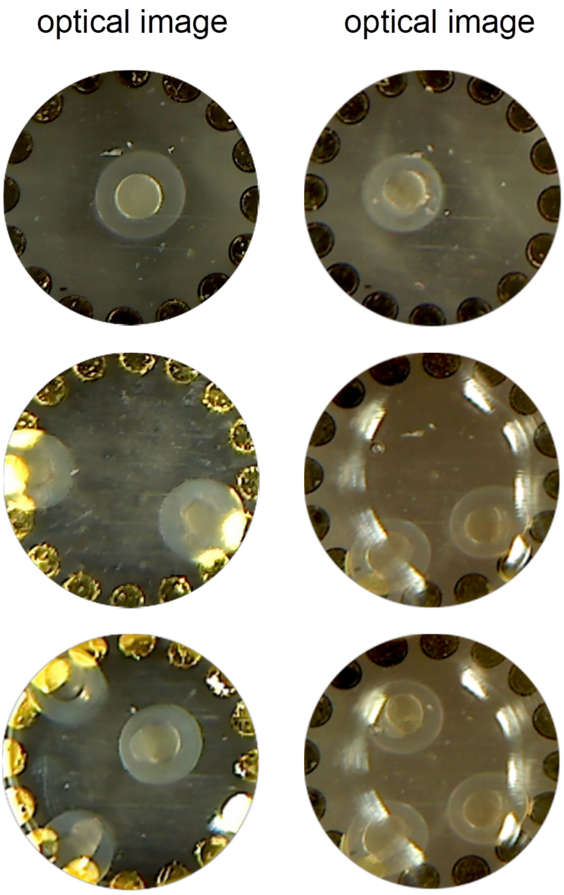

Figure S9. Original optical images of zebrafish eggs for **Figure 5** before manual software-assisted removal of reflective glare.

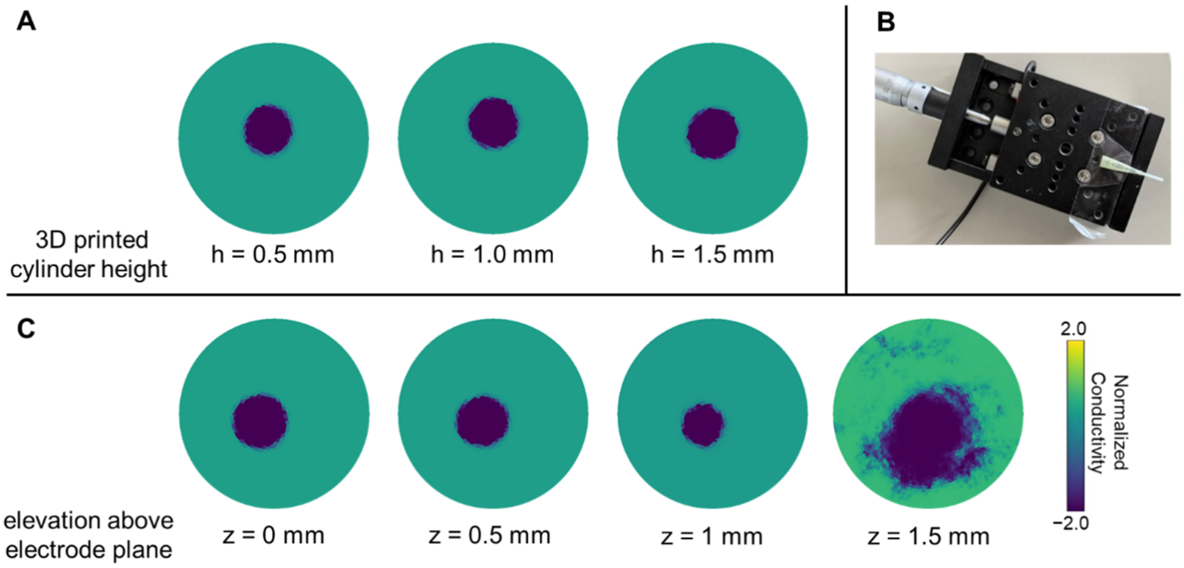

Figure S10. Evaluation of out-of-plane contributions to EIT reconstruction. (A) 3D-printed cylinders with fixed diameter of 1.5 mm and heights of h = 0.5 mm, 1.0 mm, or 1.5 mm were measured under the op–ad mode. Cylinders were positioned in contact with the electrode plane, centrally in the chamber. Corresponding CNN-based EIT reconstructions do not indicate significant differences in object size estimation. **(B)** To further interpret and investigate this, a single-axis micrometer-driven translation stage was implemented to control elevation above electrode plane: **(C)** Mounting a h = 15 mm cylinder on the aforementioned stage, we positioned it at varying vertical separation from the electrode plane z = 0 mm, 0.5 mm, 1 mm, or 1.5 mm. Here, we observe negligible changes in size estimation between the first two conditions. The reconstructed object however becomes noticeably smaller as the separation increases, with no reliable reconstruction possible at the furthest separation. We thus observe that our micro-EIT chip and reconstruction is sensitive predominantly to objects within <1 mm of the electrode plane, but with only minor sensitivity to out-of-plane variations below that range.

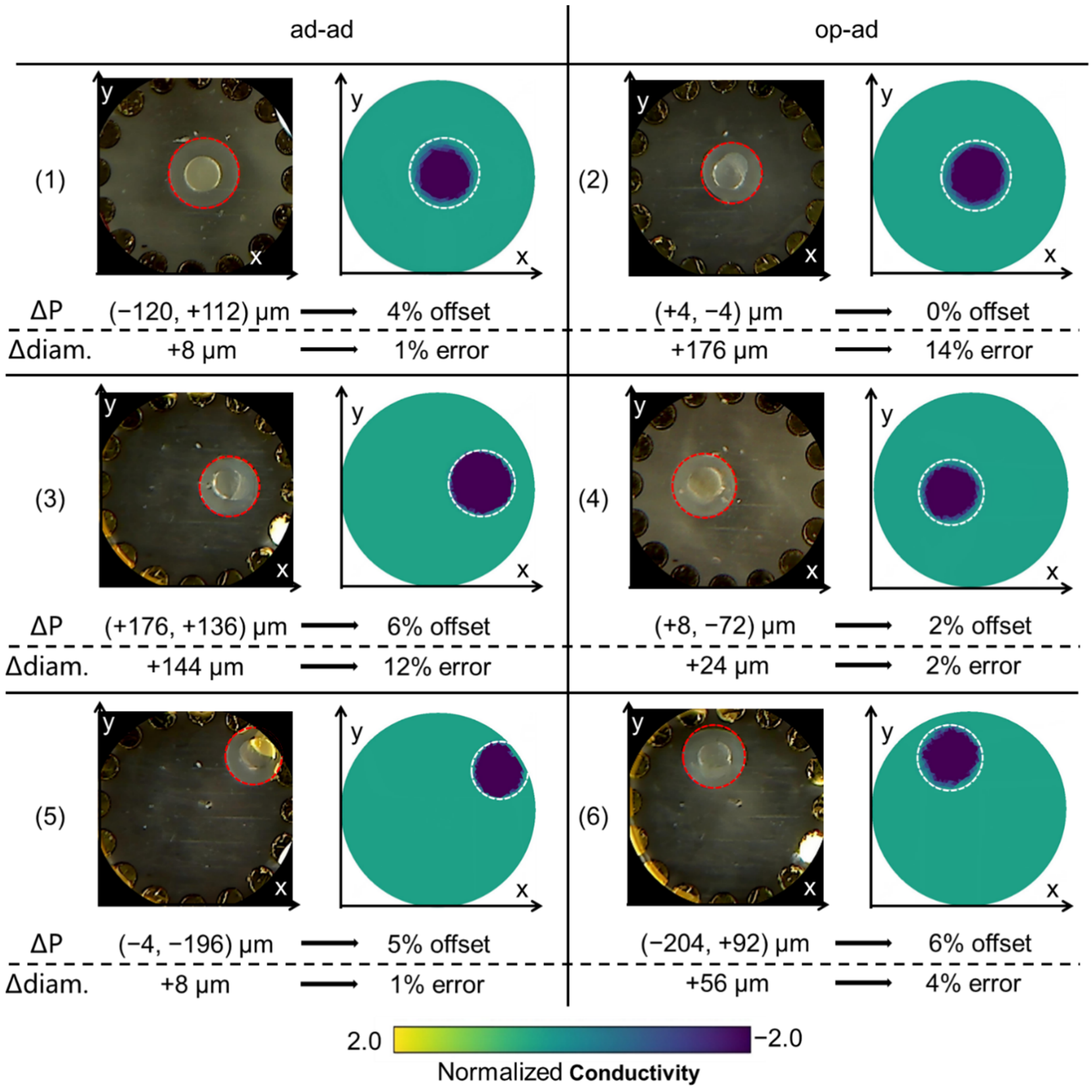

Figure S11. Accuracy of CNN reconstruction. Zebrafish eggs are positioned variously in the center, at the edge, or an intermediate position in the chamber, measured using either ad-ad or op-ad excitation-measurement mode, followed by EIT reconstruction using our CNN. We apply image segmentation to both microscope and reconstructed images to extract object center coordinates and effective circular diameters in Fiji.[13] For synthetic data, we first apply RenyiEntropy thresholding. Subsequently, we apply edge detection (i.e., differentiation) to both real images and thresholded reconstructions. For microscopy data, edge artifacts (dust, electrodes) are manually removed and low-contrast egg boundary segments manually enhanced. Finally, circular object parameters are derived by Hough Circle Transform (shown as dashed overlays). Differences in absolute position (ΔP) and diameter (Δdiam.) are computed, and normalized to the chamber diameter (4 mm) or real-image object size (~1.3 mm), respectively. Overall average relative errors of ||4%|| (position) and +6% (diameter) are highly encouraging. Given that diameter is *consistently* over-estimated, this error could likely be halved by further segmentation optimization (available thresholding algorithms in ImageJ consistently either over- or under-estimated diameters). Position errors are moreover highly

pronounced with the edge-adjacent eggs (5,6), where the optical image itself suffers from possible errors due to refraction at the curved liquid surface (meniscus). For ad-ad mode, the object in relatively close proximity to multiple electrodes (3; as opposed to equidistant/central, or on top of a single electrode) exhibits the largest errors on both metrics, i.e., the reconstruction appears both larger and closer to the electrodes than in reality. This would align with the expected “near-field” sensitivity of ad-ad mode, which the op-ad pattern can alleviate (4). Indeed, op-ad mode achieves an average positioning error half that of ad-ad mode. It does yield a sizable diameter overestimation for the centrally-located egg (4), potentially reflecting its increased sensitivity in this region.

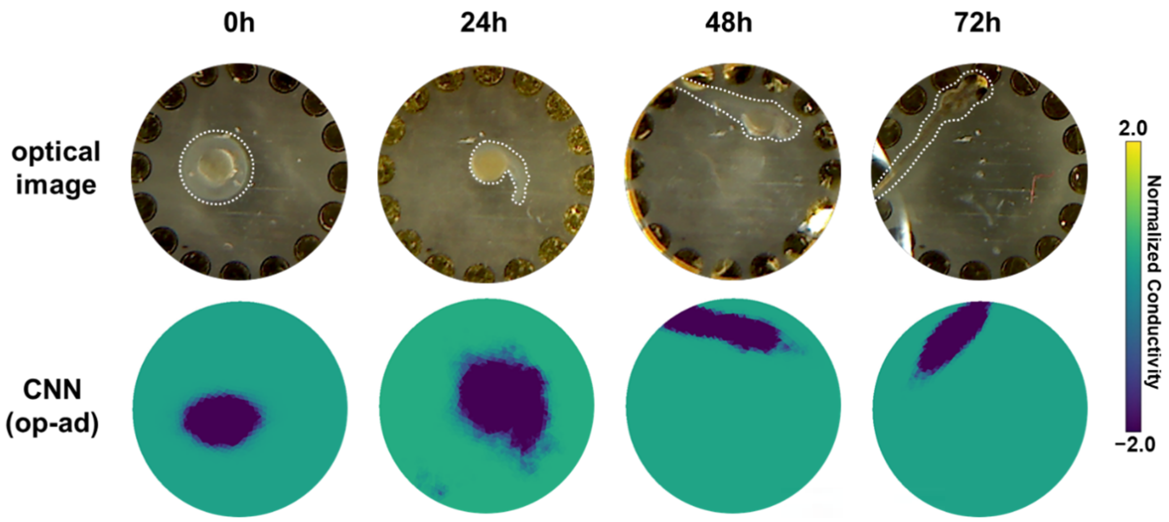

Figure S12. Model trained with circle/ellipse dataset. To evaluate whether a better shape-aligned training dataset would improve reconstruction of non-circular objects, we generated one consisting of 50% circles as otherwise employed throughout (**Table S3**), and 50% ellipses (short axis 0.1–0.4 chamber diameters; long axis 0.75–1). As expected, the corresponding CNN produced better imaging results for late pharyngula / early hatching / larval stages (48+ h), the reconstructed images more closely resembling the highly elongated biological morphology. However, the 0 h egg reconstruction appears much more elliptical than either reality or the purely-circle-trained CNNs (e.g., **Figure 5**). The reconstruction here also struggles greatly with the mid-to-late segmentation period at 48 h, capturing some of the shape but with poor boundary definition and over-estimated size. This suggests that using shape-targeted training datasets can improve imaging performance for similarly shaped samples, but at the same time can introduce additional artifacts when applied indiscriminately compared to a more general-purpose model.

References

(1) Adler, A.; Lionheart, W. R. B. Uses and Abuses of EIDORS: An Extensible Software Base for EIT. *Physiol. Meas.* **2006**, *27* (5), S25–S42. https://doi.org/10.1088/0967-3334/27/5/S03.

(2) Gabriel, S.; Lau, R. W.; Gabriel, C. The Dielectric Properties of Biological Tissues: II. Measurements in the Frequency Range 10 Hz to 20 GHz. *Phys. Med. Biol.* **1996**, *41* (11), 2251–2269. https://doi.org/10.1088/0031-9155/41/11/002.

(3) James, G.; Witten, D.; Hastie, T.; Tibshirani, R. *An Introduction to Statistical Learning: With Applications in R. Springer*; Springer, 2013. https://doi.org/10.1007/978-1-0716-1418-1.

(4) Yao J.; Wan J.; Yang L.; Liu K.; Chen B.; Wu H. Electrical characteristics of cells with electrical impedance spectroscopy. *Acta Phys. Sin.* **2020**, *69* (16), 163301. https://doi.org/10.7498/aps.69.20200601.

(5) Vorobiev, E.; Lebovka, N. I. Cell Membrane Permeabilization by Pulsed Electric Fields for Efficient Extraction of Intercellular Components from Foods. In *Pulsed Electric Fields Technology for the Food Industry: Fundamentals and Applications*; Raso, J., Heinz, V., Alvarez, I., Toepfl, S., Eds.; Springer International Publishing: Cham, 2022; pp 209–269. https://doi.org/10.1007/978-3-030-70586-2_6.

(6) Goodfellow, I.; Courville, A.; Bengio, Y. Deep Learning; Adaptive computation and machine learning; The MIT Press: Cambridge, Massachusetts, 2016.

(7) Smith, S. L.; Kindermans, P.-J.; Ying, C.; Le, Q. V. Don't Decay the Learning Rate, Increase the Batch Size. arXiv February 24, 2018. https://doi.org/10.48550/arXiv.1711.00489.

(8) Liu, C.; Chen, X.; Zhang, J.; Metz, D. ElectricSight2025/ElectricSight, 2025. https://github.com/ElectricSight2025/ElectricSight (accessed 2025-10-10).

(9) Metz, D.; Matheis, B.; Constantinou, I. Towards Micro Electrical Impedance Tomography on Chip. *SPhERe Proc. 4th Int. Symp. Pharm. Eng. Res.* **2021**. https://doi.org/10.24355/DBBS.084-202110251619-0.

(10) Harikumar, R.; Prabu, R.; Raghavan, S. Electrical Impedance Tomography (EIT) and Its Medical Applications: A Review. *Int. J. Soft Comput. Eng. IJSCE* **2013**, *3* (4), 193–198.

(11) Srivastava, N.; Hinton, G.; Krizhevsky, A.; Sutskever, I.; Salakhutdinov, R. Dropout: A Simple Way to Prevent Neural Networks from Overfitting. *J. Mach. Learn. Res.* **2014**, *15*. https://doi.org/10.5555/2627435.2670313.

(12) Atrio, À. R.; Popescu-Belis, A. Small Batch Sizes Improve Training of Low-Resource Neural MT. arXiv March 20, 2022. https://doi.org/10.48550/arXiv.2203.10579.

(13) Schindelin, J.; Arganda-Carreras, I.; Frise, E.; Kaynig, V.; Longair, M.; Pietzsch, T.; Preibisch, S.; Rueden, C.; Saalfeld, S.; Schmid, B.; Tinevez, J.-Y.; White, D. J.; Hartenstein, V.; Eliceiri, K.; Tomancak, P.; Cardona, A. Fiji: An Open-Source Platform for Biological-Image Analysis. *Nat. Methods* **2012**, *9* (7), 676–682. https://doi.org/10.1038/nmeth.2019.
